# All taxa biodiversity inventory of the Bois de Bouis estate (Var, France): a 10-year public-private partnership

**DOI:** 10.3897/BDJ.11.e103280

**Published:** 2023-08-10

**Authors:** Aurélie Lacoeuilhe, Louise Percevault, Jean Ichter, Philippe Gourdain, Katia Herard, Henri Michaud, Laurent Poncet, Thibault Ramage, Océane Roquinarc'h, Phil Withers

**Affiliations:** 1 PatriNat (OFB/MNHN/CNRS/IRD), Paris, France PatriNat (OFB/MNHN/CNRS/IRD) Paris France; 2 Muséum national d'Histoire naturelle, Paris, France Muséum national d'Histoire naturelle Paris France; 3 Conservatoire Botanique National Méditerranéen de Porquerolles, Hyères, France Conservatoire Botanique National Méditerranéen de Porquerolles Hyères France; 4 Unaffiliated, Concarneau, France Unaffiliated Concarneau France; 5 Unaffiliated, Sainte Euphémie, France Unaffiliated Sainte Euphémie France

**Keywords:** Mediterranean Region, biodiversity, habitat, conservation, France, ATBI, hotspot

## Abstract

**Background:**

This data paper describes the results of a 10-year scientific investigation of a biodiversity-rich private golf estate in south-eastern France in partnership with PatriNat (Office français de la biodiversité/Centre national de la recherche scientifique/Muséum national d'Histoire naturelle, Institut de Recherche pour le Développement). In total, 3,160 species and subspecies, including 1,796 arthropods and 1,049 flora, were inventoried and 65 habitat types were surveyed and mapped. This project is the first All taxa biodiversity inventory (ATBI) in a private property in France with all information available in open data.

**New information:**

The 20 datasets of fauna, flora, lichens and habitat types from the Bois de Bouis estate are now publicly available. Between 2012 and 2022, more than 22,000 occurrences were recorded, checked and published in the INPN information system. All this information is available in open access in the French portal OpenObs, operated by PatriNat and in the Global Biodiversity Information Facility (GBIF). This data paper provides an overview of the project, its main results and its contribution to the French National Inventory of Natural Heritage (INPN).

This data paper presents a list eight species never previously recorded to France; three Hymenoptera: *Charitopesleucobasis* Townes, 1983 (Ichneumonidae), *Dryinustussaci* Olmi, 1989 (Dryinidae) and *Sparasionmunitus* Kozlov & Kononova, 1990 (Sparasionidae) and five Diptera: *Clusiodesapicalis* (Zetterstedt, 1848) (Clusiidae), *Dicraeusvagans* (Meigen, 1838) (Chloropidae), *Stilponintermedius* Raffone, 1994, *Stilponsubnubilus* Chvala, 1988 and *Tachydromiaundulata* (Strobl, 1906) (Hybotidae).

It also includes a table comparing the project to 18 All-taxa biodiversity inventories in France and Belgium and published for the first time.

## Introduction

The Bois de Bouis estate is located in one of the hotspots of biodiversity in the Mediterranean Region (Médail and Quézel 1999), in the heart of the Plaine des Maures and partly integrated into the Plaine des Maures National Nature Reserve. It has a surface of 955 hectares including 829 hectares of semi-natural spaces open to the public and 126 hectares fenced golf course with a hamlet.

In 2007, the owner created a corporate foundation, Fondation d’Entreprise du Golf de Vidauban pour l’Environnement (FEGVE), to take care of the management of the Bois de Bouis estate. In 2011, the FEGVE and PatriNat (Office français de la biodiversité (OFB) Centre national de la recherche scientifique (CNRS)/Muséum national d'Histoire naturelle (MNHN)/Institut de Recherche pour le Développement (IRD)) began a collaboration to characterise the biodiversity of the Bois de Bouis estate and the Vidauban golf course (Delzons and Rault 2013a, Roquinarc'h et al. 2018, Roquinarc'h et al. 2019). In the context of biodiversity erosion (Cowie et al. 2022), this kind of partnership is all the more important because it improves general knowledge of biodiversity, informs owners and stakeholders of the ecological processes at stake and provides relevant technical and scientific support for management and decision-making.

**Figure 1.**
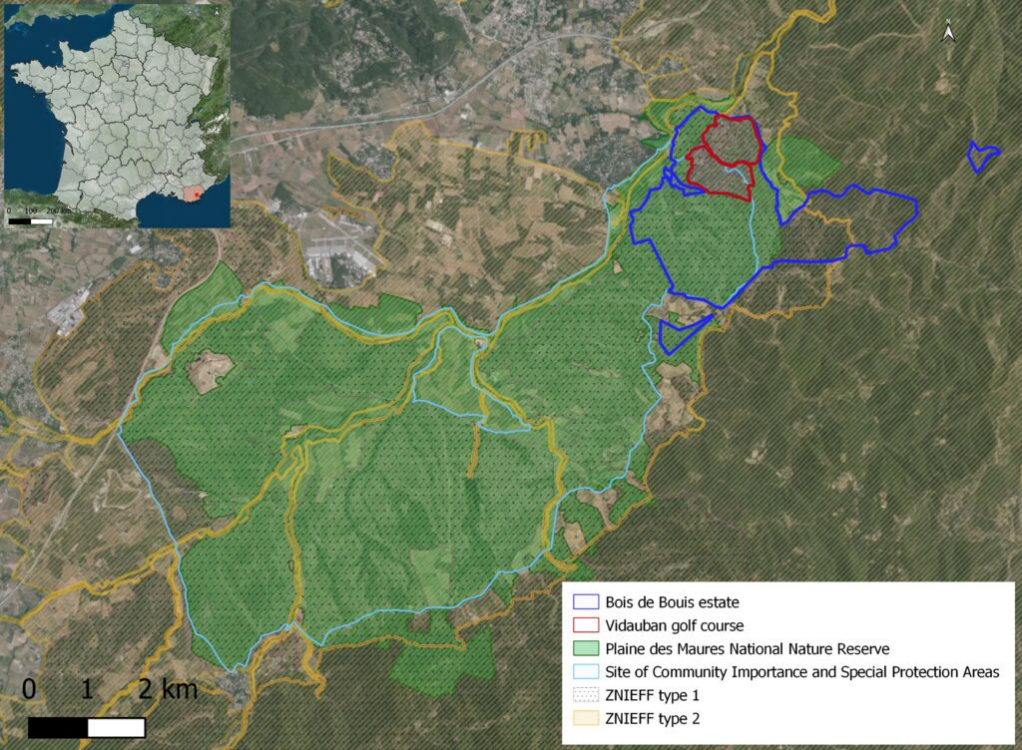
Map of the Bois de Bouis estate and its environment. Map describing the ecological environment (biodiversity area) in which the Bois de Bouis estate and the golf course are integrated. ZNIEFF type 1 and 2: Natural zone of ecological, faunistic and floristic interest.

The site's responsibility in terms of nature conservation is major for various reasons. The estate is included in different protected areas, such as Natura 2000 (Site of Community Importance n°FR9301622 and Special Protection Areas n°FR9310110) and a National Nature Reserve (n°FR3600171) and in four areas of ecological importance, ‘natural zone of ecological, faunistic or floristic value’ (Zone Naturelle d'Intérêt Écologique, Faunistique et Floristique - ZNIEFF) which is in France a natural area, regionally known for its remarkable ecological characteristics (type 1 n°930020473, type 2 n°930012553, type 2 n°930012516 and type 2 n°930020307, see Lepareur et al. (2022)) (See Fig.1). The estate also hosts habitats and species of community interest (e.g. siliceous slabs, temporary Mediterranean ponds, Serapia grasslands, *Isoetesvelata* and *I.durieii*) and IUCN Red List threatened species, such as the ocellated lizard (*Timonlepidus* (Daudin, 1802)) and the Hermann's tortoise (*Testudohermanni* Gmelin, 1789). For the latter, the Plaine des Maures population is of European importance (Couturier et al. 2014). The discovery of a population of endemic land snail *Urticicolasuberinus* (Bérenguier, 1882) in 2016 was a contribution to the site ecological importance (Léonard et al. 2016).

The objective of the partnership between PatriNat and the foundation was to tend towards a comprehensive inventory of the natural heritage to improve the management of both natural and golf areas. A biodiversity management plan was drawn up in 2016 for the estate as a continuation of the nature reserve management plan (Gourdain et al. 2017).

Fig. 1 presents a map of the Bois de Bouis estate and its environment.

## General description

### Purpose

One of the objectives of the programme is to make the information available to a wide public and to ensure it is always accessible and evolving. Experience from other AFTBIs shows that data management is often underestimated and information loss is a common issue (Langdon et al. 2006, Deharveng et al. 2015, Ichter et al. 2022).

Although the quality of the information was an important issue since the beginning of the project, the partners identified the need to allow extra resources for data quality and dissemination. All data and metadata are now structured according to the standards of the national information system for biodiversity (SINP) and the Global Biodiversity Information Facility (GBIF).

### Additional information

Before the publication of the datasets, we performed a quantitative and qualitative assessment of all data (see Quality control), an update of metadata and harmonisation of the information and we invited experts to pass on new species identification and relevant information.

The long-term preservation of data is guaranteed both technically and scientifically by PatriNat (OFB/MNHN/CNRS/IRD). According to the French Environmental Code, the MNHN is responsible for the scientific implementation of the national biodiversity inventory (INPN) and the OFB for its technical organisation.

## Project description

### Personnel

About 92 people actively participated in the project, mostly field biologists, but also taxonomists in their laboratories in France and abroad (see Acknowledgements). As part of the partnership, a full-time project manager was recruited by the MNHN to coordinate the various studies and the fieldwork, exchange with the stakeholders including the partners, manage the data and write the scientific and technical reports. A professional entomologist was tasked with sorting the material from continuous sampling methods (i.e. entomological traps) and sending specimens to the specialists. The preparation of the data paper was an opportunity for a qualitative and quantitive update of the different datasets involving staff from PatriNat and an independent consultant.

### Study area description

The Bois de Bouis estate is located in south-eastern France in a natural region called the Plaine des Maures (WGS84 decimal coordinates 43.3840, 6.4700) (see Fig. 1). Since 2009, the perimeter of the Plaine des Maures National Nature Reserve also includes part of the estate (460 hectares).

The Plaine des Maures is a sandstone depression of 13,000 hectares separating the crystalline Provence to the south from the limestone Provence to the north. The landscape is dominated by a vast mosaic of sclerophyllous shrublands, forests of stone pine (*Pinuspinea* L., 1753), cork oak (*Quercussuber* L., 1753) and evergreen oak *(Quercus ilex* L., 1753) and rocky outcrops in the continuity of the massif des Maures mountain range. The north-western part is mostly covered by agricultural land (mostly viticulture) and urban areas with significant road and rail networks. This alluvial plain is also characterised by a rich, yet seasonal, hydrographic network due to siliceous and impermeable soils, semi-arid climate, low topography and strong winds. The area is subjected to high wildfire frequency (Couturier et al. 2014).

**Figure 2.**
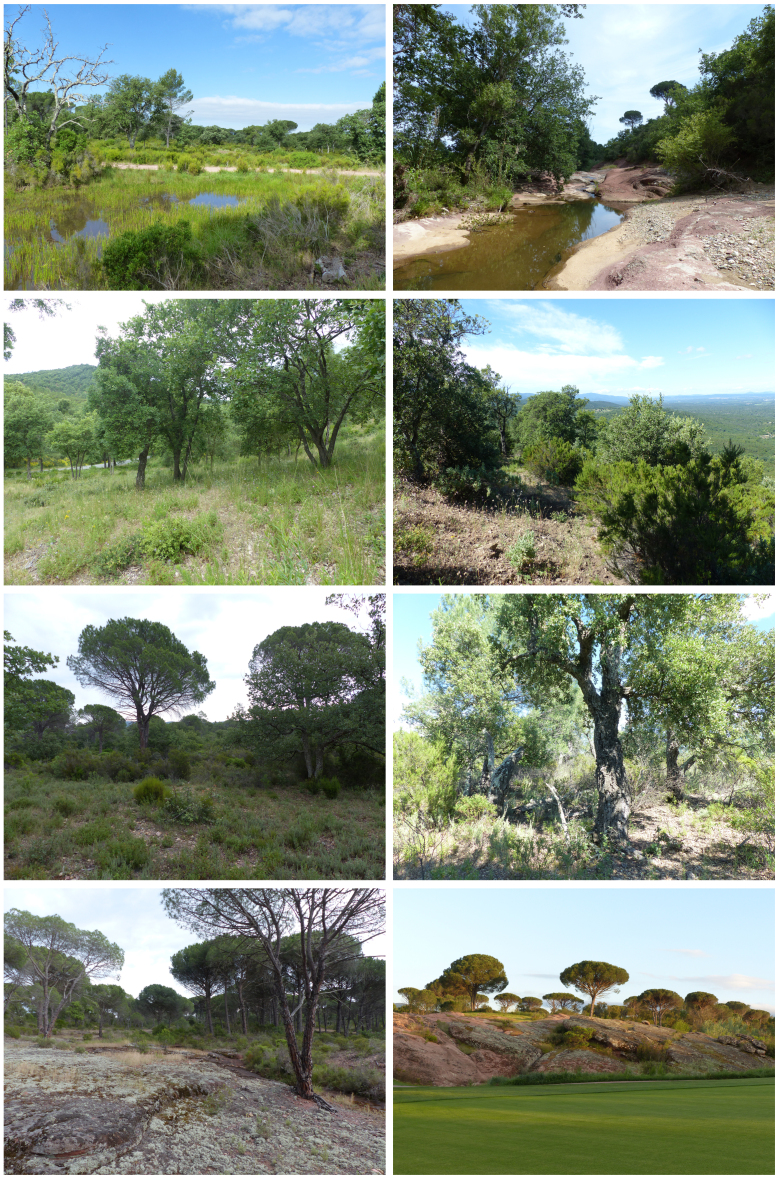
Main habitats of the Bois de Bouis estate (Author: A. Lacoeuilhe). Legend (left-to-right, top-to-bottom): C1.6 Temporary lakes, ponds and pools; C2.5 Temporary running waters; E1.81 Mediterranean therophytic siliceous grassland; F5.21 High maquis; F5.251 Central Mediterranean lavender maquis; G2.1111 Provençal cork-oak woodland; H3.62 Sparsely vegetated weathered rock and outcrop habitats; J4.6 Pavements and recreation areas.

The Bois de Bouis estate is composed of different types of natural, semi-natural and artificial habitats (see Fig. 2 and Chapter habitat type mapping).

Amongst the 126 hectares of golf course, 30 hectares are dedicated to playing areas. The tees (starting point), fairways and greens (with the hole marked with a flag) make up the real areas for playing golf. These are grassy areas, often monospecific and generally unfavourable to biodiversity. The turf is mowed at different heights depending on the areas of the course: the greens require daily maintenance while the roughs can be much less maintained. Of these 30 hectares, around 6 hectares of high-roughs have been converted to grassland since 2017. The golf course is made up of 18 holes. The other areas of the golf course correspond to a hamlet, a storage and maintenance area and semi-natural habitats.

### Design description

For well-known groups, the objective of the project was to achieve a comprehensive survey. Amphibians, birds, butterflies, dragonflies (Barbarin 2008), orthopters, reptiles, vascular flora (Michaud and Offerhaus 2016) and habitats were intensively sampled in the Bois de Bouis estate throughout the 10 years of the project using state-of-the-art methods (see Sampling methods).

The second target of the project was less-known taxonomical groups where one or several experts were available for the fieldwork at optimal observation dates: algae (Couté and Noël 2019, Noël and Couté 2020), lichens (Poncet et al. 2018, Poncet 2019), freshwater species (Desjonqueres 2016), Myriapodes (Geoffroy 2016), gasteropods (Léonard et al. 2016), hemipters (Dusoulier 2016), Coleoptera (Horellou 2015), Collembols (Deharveng and Bedos 2015), Spiders (Hervé 2012, Hervé 2014, Bosmans and Hervé 2015) and lepidoptera (Bachelard 2008, Bachelard 2009). For those groups, the objective was not a complete survey, but a reliable overview of the communities, based on surveyor’s experience.

For flying insects, interception traps (see Sampling methods) were set up. They allowed us to complete the fieldwork for some groups (e.g. coleoptera and lepidoptera) and provide material for taxonomists who did not take part in the inventories (e.g. diptera and hymenoptera) (Daugeron 2014, Ramage 2016, Rousse 2016, Whiters 2016, Tillier 2019, Quindroit 2020). A combination of standardised (i.e. trapping campaign) and opportunistic surveys is considered one of the most efficient methods to assess flying insect diversity (Deharveng et al. 2015).

An ecological assessment method, the Ecological Quality Index, was also used. As shown by the dataset, seven studies were performed from 2012 to 2021 on different parts of the Bois de Bouis to gather information for management and decision-making (Delzons and Rault 2013a, Delzons and Rault 2013b, Rault and Delzons 2014, Gourdain et al. 2017, Roquinarc'h et al. 2022). The protocol is a transect survey of reptiles, butterflies, birds, amphibians, dragonflies, vascular flora and natural habitats across the natural habitats of the site. One study involved 6 days of fieldwork over four campaigns (including two dawns and one evening) from early spring to late summer in a year. The transect survey is adapted to cover the different habitats of the study site and is repeated on each visit (different from one site to another). This method is designed to allow reproducibility for future assessments (Delzons et al. 2013). It is a semi-standardised protocol because some aspects are standardised and others are adapted according to the context (more details: https://iqe-patrinat.mnhn.fr and https://iqe-patrinat.mnhn.fr/wp-content/uploads/sites/12/2022/06/PATRINAT_ZOOM_IQE_ENG.pdf).

### Funding

The project was funded by the Fondation d’Entreprise du Golf de Vidauban pour l’Environnement (FEGVE). This corporate foundation was created in 2007 to better understand and protect the biodiversity of the Bois de Bouis estate and, more generally, to contribute to the sustainable development of the Plaine des Maures and the Massif des Maures through nature conservation actions, the production of scientific knowledge and environmental education.

## Sampling methods

### Sampling description

**Figure 3.**
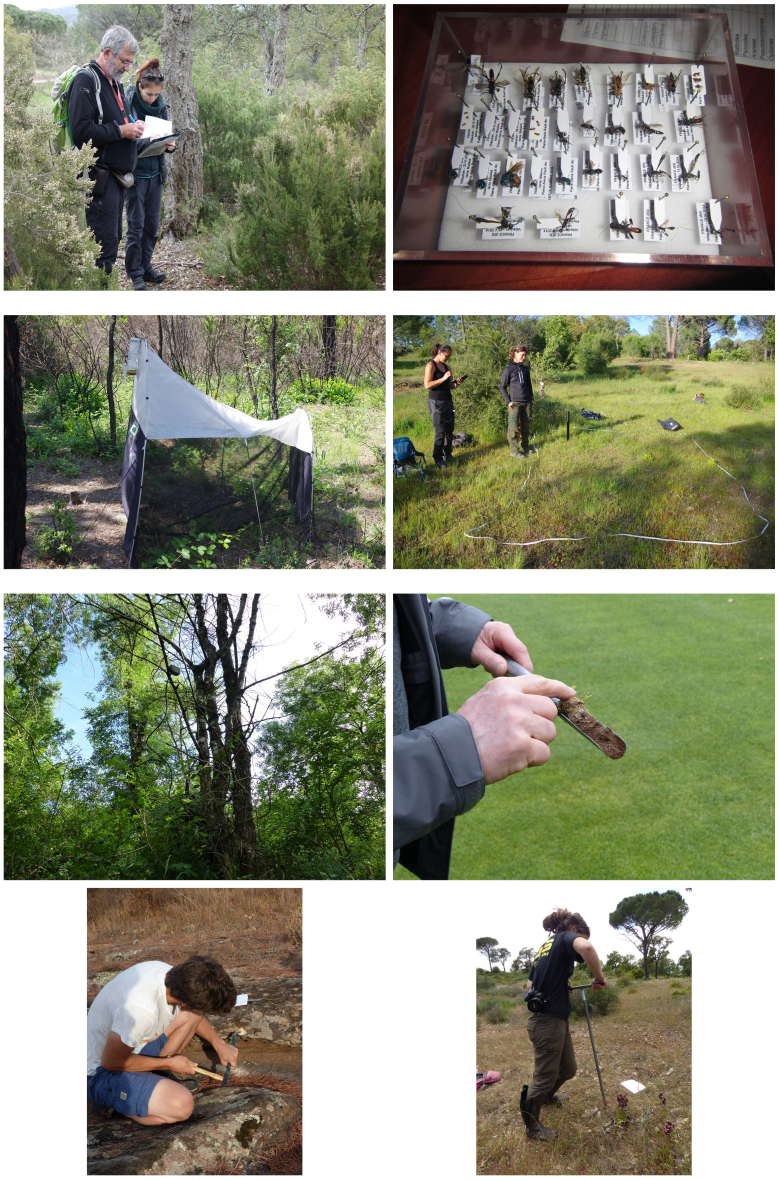
Sampling methods and techniques (Authors: photos 1 to 4 P. Gourdain under CC BY 4.0) and photos 5 to 8 A. Lacoeuilhe under CC BY 4.0). Legend (left-to-right, top-to-bottom): from top left to bottom right 1) Botanical survey; 2) Insect collection; 3) Malaise tent for insect sampling; 4) Quadrat method for vegetation monitoring; 5) SM4BAT Ultrasonic Bat Detector and Recorder; 6) Nematod sampling; 7) Saxicolous lichen sampling; 8) Serapia grassland monitoring.

Sampling methods and techniques are varied and depend on the target and the type of habitat (Fig. 3). The most frequent techniques for collecting biodiversity data involves visual and auditive contact (e.g. 10 birds count or Orthoptera inventories using transects (Lacoeuilhe et al. 2020)).

The invertebrates provide the most diverse sampling techniques: entomological net, light traps, sieving of litter, mowing of vegetation, the beating of trees and passive interception traps. Two malaise tents were placed between 28 April and 30 June 2014 and from 08 April to 20 July 2015. The first trap was set in an open area on the golf course and the second in undergrowth in the eastern part of the Bois de Bouis estate. Samples were preserved in alcohol to facilitate their management and their sending to specialists. Some specimens were identified by DNA barcoding. Around 28,000 specimens were collected and sorted.

Others specific methods were employed, such as plankton nets and sampling bottles for algae, ultrasonic bat detectors and recorder (SM2, SM4BAT and Audiomoth; Defrancq (2020), Defrancq and Lacoeuilhe (2020)), capture-recapture sampling of Hermann's tortoise (Boitier and Teynié 2007, Boitier 2008, Boitier 2010, Rault 2015), soil sampling and then laboratory study of Nematodes (Sager 2019) and vegetation monitoring plots. The habitat map was produced using vegetation surveys and a GIS software (QGIS).

The sampling methods are included in the datasets' metada using the national protocols and methods repository CAMPANULE (Ichter et al. 2014b).

### Quality control

The ATBI Bois de Bouis database is managed by PatriNat in the framework of the National Inventory of Natural Heritage (INPN). This information system guarantees the traceability of data and authorship and normalised standards of data and metadata.

A high level of data quality was expected at all stages of the programme, especially in terms of accuracy and precision (Chapman 2005). The species have been directly identified by experts or the data have been checked by experts, which guarantees a good level of reliability of the identifications.

Before data publication, two types of controls were performed. The first category of control is compliance with SINP standard formats. The data must be compliant with both physical and conceptual aspects: mandatory fields, required formats, repositories (including geographical and taxonomical, see Taxonomic coverage), classifications and lists of values. The second category of control is consistency to ensure logical compatibility within the data, the metadata and between the data and the metadata. For instance, the observation start date must be less than or equal to the observation end date.

## Geographic coverage

### Description

The Bois de Bouis estate is located in the commune of Vidauban (Post Code: 83350) in the Var Department in the Provence-Alpes-Côte d'Azur Region. The estate covers 955 hectares to the northeast of the Plaine des Maures. Most of the estate is a lowland, but the eastern part includes the foothills of the massif des Maures. Elevation ranges from 39 m to 320 m a.s.l.

### Coordinates

43.3644 and 43.403 Latitude; 6.5081 and 6.4458 Longitude.

## Taxonomic coverage

### Description

The objective of the inventory is to have a comprehensive survey of the well-known biological groups (plants, birds, mammals, amphibians, reptiles, butterflies, dragonflies and Orthoptera) and at least a preliminary inventory of less-known groups such as Aranea (spiders and pseudoscorpions), myriapods, lichens, snails, nematods and insects (i.e Blattodea, Coleoptera, Collembola, Diptera, Hemiptera, Heteroptera, Hymenoptera, Thysanoptera).

**Figure 4.**
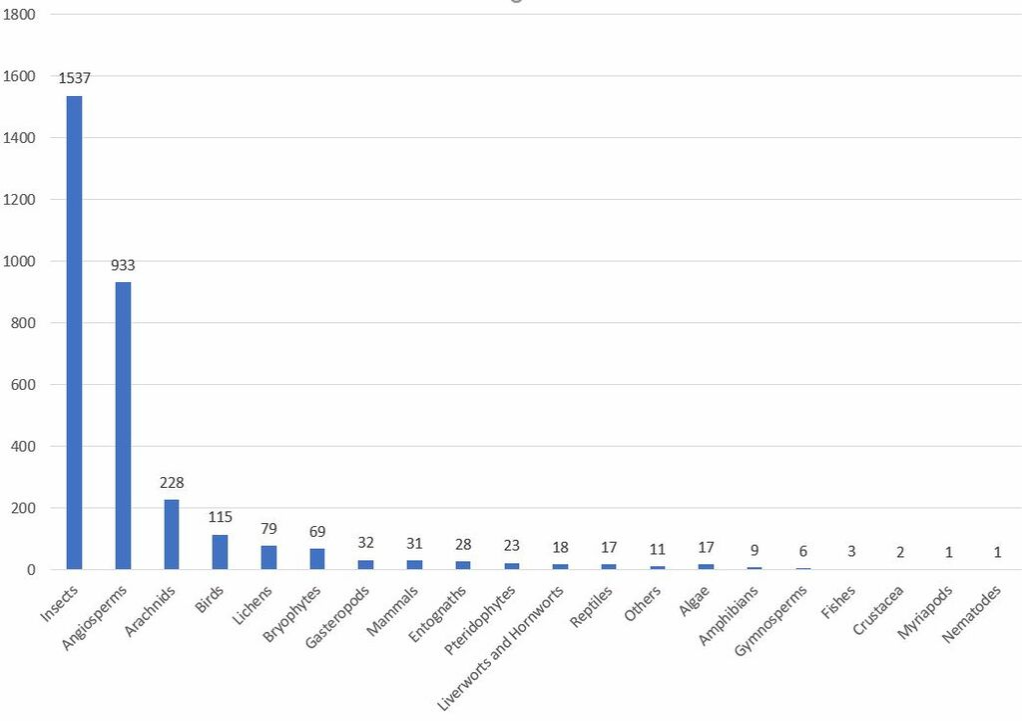
Taxonomic coverage of the inventory: number of species and subspecies per group.

**Figure 5.**
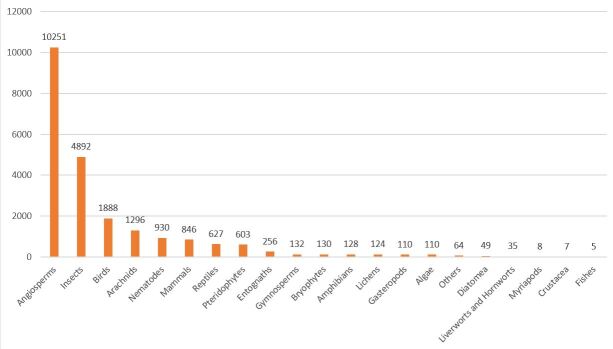
Taxonomic coverage of the inventory: numbers of data per group.

Insects represent the largest group in terms of species number (1537 species; Fig. 4) and the second in terms of data records (4892 records; Fig. 5) due to the diversity of sampling techniques either active by professional entomologists or passive with two Malaise tents.

The vascular plants are the second group for species number (933 species; Fig. 4) and the first for data records (10251 records; Fig. 5). This result is explained by the number and the variety of inventories: botanical inventories, vegetation survey and plant species monitoring.

The scientific names follow taxonomy according to TAXREF v15 (Gargominy et al. 2021). TAXREF is the national repository for flora, fauna and fungi of metropolitan France and Overseas Territories operated and managed by the Muséum national d'Histoire naturelle. Based on the most recent publications and a community of taxonomists, TAXREF assigns a unique, unambiguous and (whenever possible) consensual scientific name to all species occurring in France. The repository is constantly updated and a new version is published every year. The Bois de Bouis ATBI contributed to TAXREF with eight species not known to France prior to the inventories.

### Taxa included

**Table:** 

Rank	Scientific Name	
kingdom	Fungi	
kingdom	Plantae	
kingdom	Animalia	
phylum	Chordata	
phylum	Arthropoda	
class	Entognatha	
class	Arachnida	
class	Malacostraca	
class	Insecta	

## Temporal coverage

**Data range:** 1990-1-01 – 2021-7-29.

### Notes

The partnership between the MNHN and the FEGVE started in 2012 and ended in 2022. Most of the data were recorded during this period under the coordination of PatriNat. The datasets also include older bibliographic data (1990-2009) entered during the project, such as an entomological survey by Société d'histoire naturelle Alcide-d'Orbigny (SHNAO) in 2008-2009 (Bachelard 2008, Bachelard 2009).

**Figure 6.**
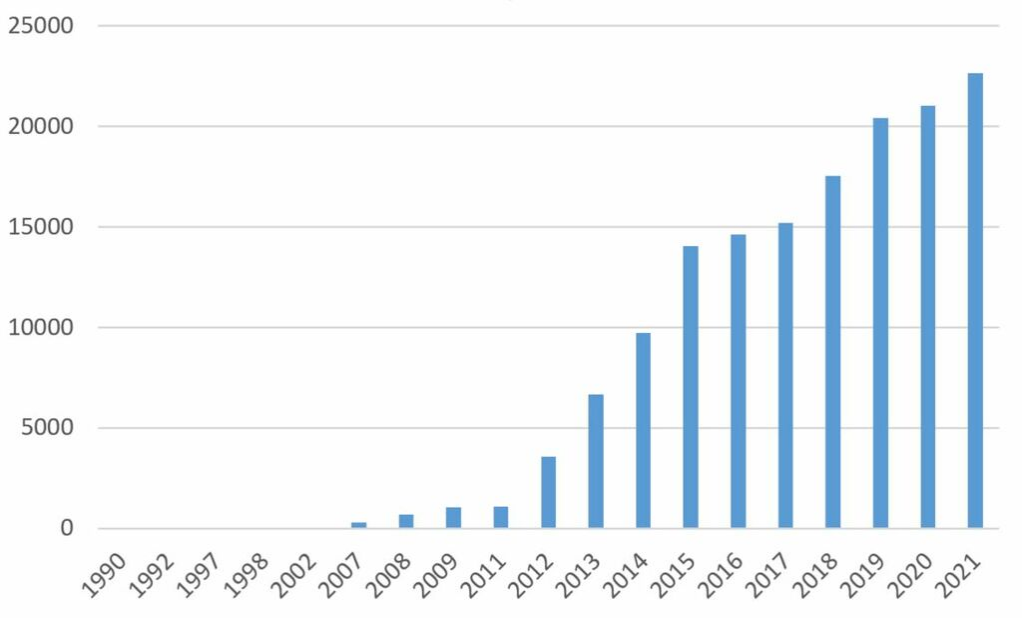
Cumulative number of data records by sample date from 1990 (1 data record) to 2021 (22654 data records). Most data records were collected in the field as part of the partnership between 2012 and 2021.

Fig. 6 shows a cumulative number of data according to the sample date. Data collected before 2012 are considered historical data provided by the FEGVE on the estate. Data collected from 2012 to 2021 are part of the partnership between FEGVE and PatriNat. In 2016 and 2017, fewer field days were performed due to data assessment and partnership renewal. In 2020 and 2021, Covid-19 limited fieldwork and, therefore, the number of data records.

## Usage licence

### Usage licence

Creative Commons Public Domain Waiver (CC-Zero)

## Data resources

### Data package title

ATBI Domaine de Bouis (Var, France)

### Number of data sets

20

### Data set 1.

#### Data set name

Inventaire général de la biodiversité (IGB/ATBI) Domaine Bois de Bouis - Données algues d'eau douce 2018-2019/All Taxa Biodiversity Inventory (ATBI) of Bois de Bouis estate - Freshwater algae data 2018-2019.

#### Data format

Darwin Core Archive

#### Download URL

https://doi.org/10.15468/b7uxaq

#### Description

This dataset was acquired in the framework of the partnership between the Fondation d'Entreprise du Golf de Vidauban pour l'Environnement (FEGVE) and PatriNat (Muséum national d'Histoire naturelle/Office français de la biodiversité/Centre national de la recherche scientifique) as part of an All Taxa Biodiversity Inventory (ATBI) of a biodiversity-rich private golf estate in south-eastern France (Vidauban, Var). This dataset contains freshwater algae data acquired between 2018 and 2019.

**Data set 1.** 

Column label	Column description
occurrenceID	An identifier for the occurrence (as opposed to a particular digital record of the occurrence).
basisOfRecord	The specific nature of the data record.
eventDate	The date when the event was recorded (dd/mm/yyyy).
recordedBy	A list (comma separated) of names of people responsible for recording the original occurrence. The primary collector or observer is listed first.
identifiedBy	A list (comma separated) of names of people who assigned the taxon to the subject.
datasetID	An identifier for the set of data. May be a global unique identifier or an identifier specific to a collection or institution.
kingdom	The full scientific name of the kingdom in which the taxon is classified.
class	The full scientific name of the class in which the taxon is classified.
order	The full scientific name of the order in which the taxon is classified.
scientificName	The full scientific name, with authorship and date information, if known.
taxonRank	The name of the taxonomic rank for which the taxon rank value is provided.
decimalLatitude	The geographic latitude (in decimal degrees, using the spatial reference system given in geodeticDatum) of the geographic centre of a Location. Positive values are north of the Equator, negative values are south of it. Legal values lie between -90 and 90, inclusive.
decimalLongitude	The geographic longitude (in decimal degrees, using the spatial reference system given in geodeticDatum) of the geographic centre of a Location. Positive values are east of the Greenwich Meridian, negative values are west of it. Legal values lie between -180 and 180, inclusive.
geodeticDatum	The ellipsoid, geodetic datum or spatial reference system (SRS) upon which the geographic coordinates given in decimalLatitude and decimalLongitude are based.
eventID	An identifier for the set of information associated with an Event (something that occurs at a place and time). May be a global unique identifier or an identifier specific to the dataset.
maximumElevationInMetres	The upper limit of the range of elevation (altitude, usually above sea level), in metres.
minimumElevationInMetres	The lower limit of the range of elevation (altitude, usually above sea level), in metres.
locality	The specific description of the place.
municipality	The full, unabbreviated name of the next smaller administrative region than county (city, municipality etc.) in which the Location occurs. Do not use this term for a nearby named place that does not contain the actual location.
country	The name of the country or major administrative unit in which the Location occurs.
countryCode	The standard code for the country in which the Location occurs.
taxonID	An identifier for the nomenclatural (not taxonomic) details of a scientific name.
originalNameUsage	The taxon name, with authorship and date information, if known, as it originally appeared when first established under the rules of the associated nomenclaturalCode.
nameAccordingTo	The reference to the source in which the specific taxon concept circumscription is defined or implied - traditionally signified by the Latin "sensu" or "sec." (from secundum, meaning "according to"). For taxa that result from identifications, a reference to the keys, monographs, experts and other sources should be given.
dateIdentified	The date on which the subject was determined as representing the Taxon.
footprintWKT	A Well-Known Text (WKT) representation of the shape (footprint, geometry) that defines the Location. A Location may have both a point-radius representation (see decimalLatitude) and a footprint representation and they may differ from each other.
county	The full, unabbreviated name of the next smaller administrative region than stateProvince (county, shire, department etc.) in which the Location occurs.
coordinateUncertaintyInMetres	The horizontal distance (in metres) from the given decimalLatitude and decimalLongitude describing the smallest circle containing the whole of the Location. Leave the value empty if the uncertainty is unknown, cannot be estimated or is not applicable (because there are no coordinates). Zero is not a valid value for this term.
modified	The most recent date-time on which the resource was changed.
dataGeneralisations	Actions taken to make the shared data less specific or complete than in its original form. Suggests that alternative data of higher quality may be available on request.
identificationVerificationStatus	A categorical indicator of the extent to which the taxonomic identification has been verified to be correct.
family	The full scientific name of the family in which the taxon is classified.
maximumDepthInMetres	The greater depth of a range of depth below the local surface, in metres (note: empty in this dataset).
minimumDepthInMetres	The lesser depth of a range of depth below the local surface, in metres (note: empty in this dataset).
locationRemarks	Comments or notes about the Location (note: empty in this dataset).
InformationWithheld	Additional information that exists, but that has not been shared in the given record (note: empty in this dataset).
institutionCode	The name (or acronym) in use by the institution having custody of the object(s) or information referred to in the record (note: empty in this dataset).
associatedReferences	A list (concatenated and separated) of identifiers (publication, bibliographic reference, global unique identifier, URI) of literature associated with the Occurrence (note: empty in this dataset).
samplingProtocol	The names of, references to, or descriptions of the methods or protocols used during an Event (note: empty in this dataset).
identificationRemarks	Comments or notes about the Identification (note: empty in this dataset).
establishmentMeans	Statement about whether an organism or organisms have been introduced to a given place and time through the direct or indirect activity of modern humans (note: empty in this dataset).
sex	The sex of the biological individual(s) represented in the Occurrence (note: empty in this dataset).
lifeStage	The age class or life stage of the Organism(s) at the time the Occurrence was recorded (note: empty in this dataset).
reproductiveCondition	The age class or life stage of the Organism(s) at the time the Occurrence was recorded (note: empty in this dataset).
associatedMedia	A list (concatenated and separated) of identifiers (publication, global unique identifier, URI) of media associated with the Occurrence (note: empty in this dataset).
behaviour	A description of the behaviour shown by the subject at the time the Occurrence was recorded (note: empty in this dataset).
occurrenceStatus	A statement about the presence or absence of a Taxon at a Location.

### Data set 2.

#### Data set name

Inventaire général de la biodiversité (IGB/ATBI) Domaine Bois de Bouis - Données bryophytes/All Taxa Biodiversity Inventory (ATBI) of Bois de Bouis estate - Bryophyte data.

#### Data format

Darwin Core Archive

#### Download URL

doi.org/10.15468/gvpgea

#### Description

This dataset was acquired in the framework of the partnership between the Fondation d'Entreprise du Golf de Vidauban pour l'Environnement (FEGVE) and PatriNat (Muséum national d'Histoire naturelle/Office français de la biodiversité/Centre national de la recherche scientifique) as part of an All Taxa Biodiversity Inventory (ATBI) of a biodiversity-rich private golf estate in south-eastern France (Vidauban, Var). This dataset contains bryophyte data acquired in 2016.

**Data set 2.** 

Column label	Column description
idem as "Inventaire général de la biodiversité (IGB/ATBI) Domaine Bois de Bouis - Données algues d'eau douce 2018-2019"	idem as "Inventaire général de la biodiversité (IGB/ATBI) Domaine Bois de Bouis - Données algues d'eau douce 2018-2019".

### Data set 3.

#### Data set name

Inventaire général de la biodiversité (IGB/ATBI) Domaine Bois de Bouis - Données de suivi Tortue d'Hermann (Testudohermanni)/All Taxa Biodiversity Inventory (ATBI) of Bois de Bouis estate - Hermann's Tortoise (Testudohermanni) monitoring data.

#### Data format

Darwin Core Archive

#### Download URL

doi.org/10.15468/x4ay8b

#### Description

This dataset was acquired in the framework of the partnership between the Fondation d'Entreprise du Golf de Vidauban pour l'Environnement (FEGVE) and PatriNat (Muséum national d'Histoire naturelle/Office français de la biodiversité/Centre national de la recherche scientifique) as part of an All Taxa Biodiversity Inventory (ATBI) of a biodiversity-rich private golf estate in south-eastern France (Vidauban, Var). This dataset contains monitoring data for Hermann's Tortoise (*Testudohermanni*).

**Data set 3.** 

Column label	Column description
idem as "Inventaire général de la biodiversité (IGB/ATBI) Domaine Bois de Bouis - Données algues d'eau douce 2018-2019"	idem as "Inventaire général de la biodiversité (IGB/ATBI) Domaine Bois de Bouis - Données algues d'eau douce 2018-2019".

### Data set 4.

#### Data set name

Inventaire général de la biodiversité (IGB/ATBI) Domaine Bois de Bouis - Données étude chiropteres 2020/All Taxa Biodiversity Inventory (ATBI) of Bois de Bouis estate - Chiroptera data 2020.

#### Data format

Darwin Core Archive

#### Download URL

https://doi.org/10.15468/payrgn

#### Description

This dataset was acquired in the framework of the partnership between the Fondation d'Entreprise du Golf de Vidauban pour l'Environnement (FEGVE) and PatriNat (Muséum national d'Histoire naturelle/Office français de la biodiversité/Centre national de la recherche scientifique) as part of an All Taxa Biodiversity Inventory (ATBI) of a biodiversity-rich private golf estate in south-eastern France (Vidauban, Var). The dataset contains bat (chiroptera) data acquired in 2020.

**Data set 4.** 

Column label	Column description
idem as "Inventaire général de la biodiversité (IGB/ATBI) Domaine Bois de Bouis - Données algues d'eau douce 2018-2019"	idem as "Inventaire général de la biodiversité (IGB/ATBI) Domaine Bois de Bouis - Données algues d'eau douce 2018-2019".

### Data set 5.

#### Data set name

Inventaire général de la biodiversité (IGB/ATBI) Domaine Bois de Bouis - Données étude pelouses à serapias 2018-2019/All Taxa Biodiversity Inventory (ATBI) of Bois de Bouis estate - Serapia grassland data 2018-2019.

#### Data format

Darwin Core Archive

#### Download URL

https://doi.org/10.15468/vnunwj

#### Description

This dataset was acquired in the framework of the partnership between the Fondation d'Entreprise du Golf de Vidauban pour l'Environnement (FEGVE) and PatriNat (Muséum national d'Histoire naturelle/Office français de la biodiversité/Centre national de la recherche scientifique) as part of an All Taxa Biodiversity Inventory (ATBI) of a biodiversity-rich private golf estate in south-eastern France (Vidauban, Var). This dataset contains data from a Serapia grassland survey acquired between 2018 and 2019.

**Data set 5.** 

Column label	Column description
idem as "Inventaire général de la biodiversité (IGB/ATBI) Domaine Bois de Bouis - Données algues d'eau douce 2018-2019"	idem as "Inventaire général de la biodiversité (IGB/ATBI) Domaine Bois de Bouis - Données algues d'eau douce 2018-2019".

### Data set 6.

#### Data set name

Inventaire général de la biodiversité (IGB/ATBI) Domaine Bois de Bouis - Données flore/All Taxa Biodiversity Inventory (ATBI) of Bois de Bouis estate - Floristic data.

#### Data format

Darwin Core Archive

#### Download URL

https://doi.org/10.15468/parzzs

#### Description

This dataset was acquired in the framework of the partnership between the Fondation d'Entreprise du Golf de Vidauban pour l'Environnement (FEGVE), PatriNat (Muséum national d'Histoire naturelle/Office français de la biodiversité/Centre national de la recherche scientifique) and the Conservatoire Botanique National Méditerranéen (CBNMed) as part of an All Taxa Biodiversity Inventory (ATBI) of a biodiversity-rich private golf estate in south-eastern France (Vidauban, Var). The dataset contains floristic data acquired between 2018 and 2019.

**Data set 6.** 

Column label	Column description
idem as "Inventaire général de la biodiversité (IGB/ATBI) Domaine Bois de Bouis - Données algues d'eau douce 2018-2019"	idem as "Inventaire général de la biodiversité (IGB/ATBI) Domaine Bois de Bouis - Données algues d'eau douce 2018-2019".

### Data set 7.

#### Data set name

Inventaire général de la biodiversité (IGB/ATBI) Domaine Bois de Bouis - Données lichens 2017-2021/All Taxa Biodiversity Inventory (ATBI) of Bois de Bouis estate - Lichen data 2017-2021.

#### Data format

Darwin Core Archive

#### Download URL

https://doi.org/10.15468/vrybab

#### Description

This dataset was acquired in the framework of the partnership between the Fondation d'Entreprise du Golf de Vidauban pour l'Environnement (FEGVE) and PatriNat (Muséum national d'Histoire naturelle/Office français de la biodiversité/Centre national de la recherche scientifique) as part of an All Taxa Biodiversity Inventory (ATBI) of a biodiversity-rich private golf estate in south-eastern France (Vidauban, Var). The dataset contains lichen data acquired between 2017 and 2021.

**Data set 7.** 

Column label	Column description
idem as "Inventaire général de la biodiversité (IGB/ATBI) Domaine Bois de Bouis - Données algues d'eau douce 2018-2019"	idem as "Inventaire général de la biodiversité (IGB/ATBI) Domaine Bois de Bouis - Données algues d'eau douce 2018-2019".

### Data set 8.

#### Data set name

Inventaire général de la biodiversité (IGB/ATBI) Domaine Bois de Bouis - Données nématodes 2018-2019/All Taxa Biodiversity Inventory (ATBI) of Bois de Bouis estate - Nematode data 2018-2019.

#### Data format

Darwin Core Archive

#### Download URL

https://doi.org/10.15468/j7ezgn

#### Description

This dataset was acquired in the framework of the partnership between the Fondation d'Entreprise du Golf de Vidauban pour l'Environnement (FEGVE) and PatriNat (Muséum national d'Histoire naturelle/Office français de la biodiversité/Centre national de la recherche scientifique) as part of an All Taxa Biodiversity Inventory (ATBI) of a biodiversity-rich private golf estate in south-eastern France (Vidauban, Var). The dataset contains nematode data acquired between 2018 and 2019.

**Data set 8.** 

Column label	Column description
idem as "Inventaire général de la biodiversité (IGB/ATBI) Domaine Bois de Bouis - Données algues d'eau douce 2018-2019"	idem as "Inventaire général de la biodiversité (IGB/ATBI) Domaine Bois de Bouis - Données algues d'eau douce 2018-2019".

### Data set 9.

#### Data set name

Inventaire général de la biodiversité (IGB/ATBI) Domaine Bois de Bouis - Données opportunistes/All Taxa Biodiversity Inventory (ATBI) of Bois de Bouis estate - Opportunistic data.

#### Data format

Darwin Core Archive

#### Download URL

https://doi.org/10.15468/w65gbj

#### Description

This dataset was acquired in the framework of the partnership between the Fondation d'Entreprise du Golf de Vidauban pour l'Environnement (FEGVE) and PatriNat (Muséum national d'Histoire naturelle/Office français de la biodiversité/Centre national de la recherche scientifique) as part of an All Taxa Biodiversity Inventory (ATBI) of a biodiversity-rich private golf estate in south-eastern France (Vidauban, Var). This dataset contains opportunistic data acquired between 2011 and 2021.

**Data set 9.** 

Column label	Column description
idem as "Inventaire général de la biodiversité (IGB/ATBI) Domaine Bois de Bouis - Données algues d'eau douce 2018-2019"	idem as "Inventaire général de la biodiversité (IGB/ATBI) Domaine Bois de Bouis - Données algues d'eau douce 2018-2019".

### Data set 10.

#### Data set name

Inventaire général de la biodiversité (IGB/ATBI) Domaine Bois de Bouis - Données pièges à interception insectes/All Taxa Biodiversity Inventory (ATBI) of Bois de Bouis estate - Insect interception trap data.

#### Data format

Darwin Core Archive

#### Download URL

https://doi.org/10.15468/48jh8f

#### Description

This dataset was acquired in the framework of the partnership between the Fondation d'Entreprise du Golf de Vidauban pour l'Environnement (FEGVE) and PatriNat (Muséum national d'Histoire naturelle/Office français de la biodiversité/Centre national de la recherche scientifique) as part of an All Taxa Biodiversity Inventory (ATBI) of a biodiversity-rich private golf estate in south-eastern France (Vidauban, Var). The present dataset contains insect data acquired between 2013 and 2015 from flight interception traps.

**Data set 10.** 

Column label	Column description
idem as "Inventaire général de la biodiversité (IGB/ATBI) Domaine Bois de Bouis - Données algues d'eau douce 2018-2019"	idem as "Inventaire général de la biodiversité (IGB/ATBI) Domaine Bois de Bouis - Données algues d'eau douce 2018-2019".

### Data set 11.

#### Data set name

Inventaire général de la biodiversité (IGB/ATBI) Domaine Bois de Bouis - Données suivi orthoptères 2017-2019/All Taxa Biodiversity Inventory (ATBI) of Bois de Bouis estate - Orthoptera monitoring data 2017-2019.

#### Data format

Darwin Core Archive

#### Download URL

https://doi.org/10.15468/9s77nz

#### Description

This dataset was acquired in the framework of the partnership between the Fondation d'Entreprise du Golf de Vidauban pour l'Environnement (FEGVE) and PatriNat (Muséum national d'Histoire naturelle/Office français de la biodiversité/Centre national de la recherche scientifique) as part of an All Taxa Biodiversity Inventory (ATBI) of a biodiversity-rich private golf estate in south-eastern France (Vidauban, Var). This dataset contains Orthoptera monitoring data acquired between 2017 and 2019.

**Data set 11.** 

Column label	Column description
idem as "Inventaire général de la biodiversité (IGB/ATBI) Domaine Bois de Bouis - Données algues d'eau douce 2018-2019"	idem as "Inventaire général de la biodiversité (IGB/ATBI) Domaine Bois de Bouis - Données algues d'eau douce 2018-2019".

### Data set 12.

#### Data set name

Inventaire général de la biodiversité (IGB/ATBI) Domaine Bois de Bouis - Données historiques/All Taxa Biodiversity Inventory (ATBI) of Bois de Bouis estate - Historical data.

#### Data format

Darwin Core Archive

#### Download URL

https://doi.org/10.15468/gz4ywu

#### Description

This dataset was acquired in the framework of the partnership between the Fondation d'Entreprise du Golf de Vidauban pour l'Environnement (FEGVE) and PatriNat (Muséum national d'Histoire naturelle/Office français de la biodiversité/Centre national de la recherche scientifique) as part of an All Taxa Biodiversity Inventory (ATBI) of a biodiversity-rich private golf estate in south-eastern France (Vidauban, Var). This dataset contains historical fauna and flora data acquired between 1990 and 2009.

**Data set 12.** 

Column label	Column description
idem as "Inventaire général de la biodiversité (IGB/ATBI) Domaine Bois de Bouis - Données algues d'eau douce 2018-2019"	idem as "Inventaire général de la biodiversité (IGB/ATBI) Domaine Bois de Bouis - Données algues d'eau douce 2018-2019".

### Data set 13.

#### Data set name

Données espèces-Expertise Indice de Potentialité Ecologique (IPE) Bois de Bouis 2015/Ecological Potential Index Bois de Bouis 2015.

#### Data format

Darwin Core Archive

#### Download URL

https://doi.org/10.15468/rs7y9p

#### Description

This dataset was acquired as part of the partnership between the Fondation d'Entreprise du Golf de Vidauban pour l'Environnement (FEGVE) and PatriNat (MNHN - OFB - CNRS). The Bois de Bouis estate is a private property of 870 ha in the heart of the Plaine des Maures in the town of Vidauban in the Var Department (83). This dataset corresponds to species data from the Ecological Potential Index (EPI) expertise in the Bois de Bouis in 2015.

**Data set 13.** 

Column label	Column description
idem as "Inventaire général de la biodiversité (IGB/ATBI) Domaine Bois de Bouis - Données algues d'eau douce 2018-2019"	idem as "Inventaire général de la biodiversité (IGB/ATBI) Domaine Bois de Bouis - Données algues d'eau douce 2018-2019".

### Data set 14.

#### Data set name

Données espèces-Expertise Indice de Qualité Ecologique (IQE) Bois de Bouis 2012/Ecological Quality Index Bois de Bouis 2012.

#### Data format

Darwin Core Archive

#### Download URL

https://doi.org/10.15468/vzgjp5

#### Description

This dataset was acquired as part of the partnership between the Fondation d'Entreprise du Golf de Vidauban pour l'Environnement (FEGVE) and PatriNat (MNHN - OFB - CNRS). The Bois de Bouis estate is a private property of 870 ha in the heart of the Plaine des Maures in the town of Vidauban in the Var Department (83). This dataset corresponds to species data from the Ecological Quality Index (EQI) expertise in the Bois de Bouis in 2012.

**Data set 14.** 

Column label	Column description
idem as "Inventaire général de la biodiversité (IGB/ATBI) Domaine Bois de Bouis - Données algues d'eau douce 2018-2019"	idem as "Inventaire général de la biodiversité (IGB/ATBI) Domaine Bois de Bouis - Données algues d'eau douce 2018-2019".

### Data set 15.

#### Data set name

Données espèces-Expertise Indice de Qualité Ecologique (IQE) Golf de Vidauban 2012/Ecological Quality Index Vidauban Golf course 2012.

#### Data format

Darwin Core Archive

#### Download URL

https://doi.org/10.15468/qtax2u

#### Description

This dataset was acquired as part of the partnership between the Fondation d'Entreprise du Golf de Vidauban pour l'Environnement (FEGVE) and PatriNat (MNHN - OFB - CNRS). The Bois de Bouis estate is a private property of 870 ha in the heart of the Plaine des Maures in the town of Vidauban in the Var Department (83). This dataset corresponds to species data from the Ecological Quality Index (EQI) expertise on the golf course in 2012.

**Data set 15.** 

Column label	Column description
idem as "Inventaire général de la biodiversité (IGB/ATBI) Domaine Bois de Bouis - Données algues d'eau douce 2018-2019"	idem as "Inventaire général de la biodiversité (IGB/ATBI) Domaine Bois de Bouis - Données algues d'eau douce 2018-2019".

### Data set 16.

#### Data set name

Données espèces-Expertise Indice de Qualité Ecologique (IQE) Golf de Vidauban 2014/Ecological Quality Index Vidauban Golf course 2014.

#### Data format

Darwin Core Archive

#### Download URL

https://doi.org/10.15468/cykff2

#### Description

This dataset was acquired as part of the partnership between the Fondation d'Entreprise du Golf de Vidauban pour l'Environnement (FEGVE) and PatriNat (MNHN - OFB - CNRS). The Bois de Bouis estate is a private property of 870 ha in the heart of the Plaine des Maures in the town of Vidauban in the Var Department (83). This dataset corresponds to species data from the Ecological Quality Index (EQI) expertise on the golf course in 2014.

**Data set 16.** 

Column label	Column description
idem as "Inventaire général de la biodiversité (IGB/ATBI) Domaine Bois de Bouis - Données algues d'eau douce 2018-2019"	idem as "Inventaire général de la biodiversité (IGB/ATBI) Domaine Bois de Bouis - Données algues d'eau douce 2018-2019".

### Data set 17.

#### Data set name

Données espèces-Expertise Indice de Qualité Ecologique (IQE) secteur Est du domaine 2013/Ecological Quality Index East Bois de Bouis 2013.

#### Data format

Darwin Core Archive

#### Download URL

https://doi.org/10.15468/f2tpby

#### Description

This dataset was acquired as part of the partnership between the Fondation d'Entreprise du Golf de Vidauban pour l'Environnement (FEGVE) and PatriNat (MNHN - OFB - CNRS). The Bois de Bouis estate is a private property of 870 ha in the heart of the Plaine des Maures in the town of Vidauban in the Var Department (83). This dataset corresponds to species data from the Ecological Quality Index (EQI) expertise in the eastern part of the estate.

**Data set 17.** 

Column label	Column description
idem as "Inventaire général de la biodiversité (IGB/ATBI) Domaine Bois de Bouis - Données algues d'eau douce 2018-2019"	idem as "Inventaire général de la biodiversité (IGB/ATBI) Domaine Bois de Bouis - Données algues d'eau douce 2018-2019".

### Data set 18.

#### Data set name

Données espèces-Expertise Indice de Qualité Ecologique (IQE) Golf de Vidauban 2021/Ecological Quality Index Vidauban Golf 2021.

#### Data format

Darwin Core Archive

#### Download URL

https://doi.org/10.15468/7ugtew

#### Description

This dataset was acquired as part of the partnership between the Fondation d'Entreprise du Golf de Vidauban pour l'Environnement (FEGVE) and PatriNat (MNHN - OFB - CNRS). The Bois de Bouis estate is a private property of 870 ha in the heart of the Plaine des Maures in the town of Vidauban in the Var Department (83). This dataset corresponds to species data from the Ecological Quality Index (EQI) expertise on the golf course in 2021.

**Data set 18.** 

Column label	Column description
idem as "Inventaire général de la biodiversité (IGB/ATBI) Domaine Bois de Bouis - Données algues d'eau douce 2018-2019"	idem as "Inventaire général de la biodiversité (IGB/ATBI) Domaine Bois de Bouis - Données algues d'eau douce 2018-2019".

### Data set 19.

#### Data set name

Inventaire général de la biodiversité (IGB/ATBI) Domaine Bois deBouis - Données mollusques continentaux/All Taxa Biodiversity Inventory (ATBI) ofBois de Bouis estate - Continental mollusc data.

#### Data format

darwin core

#### Download URL

https://doi.org/10.15468/y3q4ua

#### Description

This dataset was acquired in the framework of the partnership between the Fondation d'Entreprise du Golf de Vidauban pour l'Environnement (FEGVE) and PatriNat (Muséum national d'Histoire naturelle/Office français de la biodiversité/Centre national de la recherche scientifique) as part of an All Taxa Biodiversity Inventory (ATBI) of a biodiversity-rich private golf estate in south-eastern France (Vidauban, Var). This dataset contains mollusc data acquired between 2018 and 2019.

**Data set 19.** 

Column label	Column description
idem as "Inventaire général de la biodiversité (IGB/ATBI) Domaine Bois de Bouis - Données algues d'eau douce 2018-2019"	idem as "Inventaire général de la biodiversité (IGB/ATBI)Domaine Bois de Bouis - Données algues d'eau douce2018-2019".

### Data set 20.

#### Data set name

Habitat mapping Bois de Bouis estate.

#### Data format

geospatial vector data

#### Description

This habitat dataset was acquired as part of the partnership between the Fondation d'Entreprise du Golf de Vidauban pour l'Environnement (FEGVE) and PatriNat (MNHN - OFB - CNRS). The Bois de Bouis estate is a private property of 870 hectares in the heart of the Plaine des Maures in the town of Vidauban in the Var Department (83). Habitat survey was carried out between 2014 and 2021 for the whole site at a scale of 1:5000 using EUNIS typology.

**Data set 20.** 

Column label	Column description
id_evenement	Unique identifier of the event.
id_sinp_evenement	Unique identifier of the event. It takes the form of a UUID (Universally unique identifier).
date_obs_debut	Starting date of the observation period.
date_obs_fin	End date of the observation period.
cd_sig	Unique identifier of the geometry.
commentaire	commentaire Free field for any additional information indicative of the observation event.
echelle_numerisation	The scale of geographic information digitisation. If the polygon is viewed at a precision other than the one presented here, the precision of the geographic information may be affected.
est_mosaique	Used to indicate the presence of several habitats within the same station (habitat mosaic or complex).
identite_observateur	Organisation(s) to which the habitat observer(s) is/are attached.
nature_objet_geo	Nature of the geographical object (location transmitted). Used to indicate whether the habitat or habitat complex observed is stationary (it is present on the whole of the geographical object) or inventory (it is found on a given part of the geographical object without further precision).
type_geom	Type of geometry of the habitat observation event.
id_sinp_jdd	Permanent and unique identifier of the dataset metadata record in the SINP to which the observation data belongs.
id_jddid_jdd	Another unique identifier of the metadata record of the dataset in the SINP to which the observation data belongs.
id_obs_habitat	Unique identifier of the habitat observation.
id_sinp_habitat	Unique identifier of the observed habitat. It takes the form of a UUID (Universally unique identifier).
id_origine_habitat	Unique identifier of the observation in the producer database where the original data are stored and initially managed.
nom_cite	Name of the habitat originally cited by the observer.
code_hab_cite	Code of the habitat originally cited by the observer or by the determiner (when it is not the same person). Code assigned to the habitat unit in the typology used by the data producer to describe the habitat observation and recorded in the source database. It should not be confused with the cdHab, which is the unique identifier of the habitat unit in the HABREF repository (when this habitat unit is included).
nom_typo	Typology to which the habitat unit is attached. This information is particularly useful in the case where the typology does not appear in the national reference system. (HABREF).
cd_hab	Unique identifier of the housing unit within the HABREF repository. This identifier is named CD_HAB in the HABREF repository.
nom_habitat	Name of the habitat in the HABREF repository, when the cd_hab field is filled in.
technique_collecte	Collection technique used to generate the observation.
recouvrement	Percentage of habitat overlapping in relation to the geographical unit. In the case of a habitat mosaic, it is used to estimate the share of the habitat within the mosaic.
abondance_habitat	Relative abundance of the habitat in relation to the geographical unit. Used to enter a relative cover, when an exact cover cannot be estimated.
commentaire	Free field for any additional indicative information on the observed habitat.
identite_determinateur	Identity (surname, first name) of the person(s) who carried out the habitat determination.
organisme_determinateur	The organisation to which the person(s) who carried out the habitat determination is/are attached.

## Additional information

### Key outcomes of the inventories

Thanks to the project, 3,160 species and subspecies are now known from the Bois de Bouis estate. In total, 22,654 data sources were recorded, checked and published in the INPN information system. All this information is now available in open access in the GBIF web site (see Data resources) and in the INPN via the OpenObs portal.

The inventories added 77 species and one subspecies for which no occurrences were previously recorded in the national inventory (INPN). At regional level, the project published 206 species and six subspecies for which no occurrences were previously recorded in the Provence-Alpes Côte d'Azur region, 457 species and 15 subspecies new for the Var Departement and 1618 species and 58 subspecies new for the Vidauban commune.

Table 1 presents the number of species occurring in the Bois de Bouis estate and its proportion compared to species known to occur in metropolitan France.

### New species to France and taxonomical advances

The two Malaise traps set in the Bois de Bouis estate in 2014 and in 2015 by Pierre-Alexis Rault allowed the collection of 28,064 arthropod specimens of which 19,326 specimens were sent to taxonomists. Most specimens belong to Hymenoptera (10139) and Diptera (8860).

Although a large number of specimens remain unidentified at the species level, eight species from the Bois du Bouis estate are here reported for the first time from France:

- the three Hymenoptera identified by Pascal Rousse, Massimo Olmi and Thibault Ramage are *Charitopesleucobasis* Townes, 1983 (Ichneumonidae), *Dryinustussaci* Olmi, 1989 (Dryinidae) and *Sparasionmunitus* Kozlov & Kononova, 1990 (Sparasionidae) ;
- the five Diptera identified by Phil Whiters are *Clusiodesapicalis* (Zetterstedt, 1848) (Clusiidae), *Dicraeusvagans* (Meigen, 1838) (Chloropidae), *Stilponintermedius* Raffone, 1994, *Stilponsubnubilus* Chvala, 1988 and *Tachydromiaundulata* (Strobl, 1906) (Hybotidae).

Several specimens belong to rare species known from very few locations in France, such as *Cercopisarcuata* Fieber, 1844 (Cercopidae) or *Onychopterocheilushellenicus* (Morawitz, 1885) (Vespidae).

The specimens collected, sorted and sent to specialists helped to improve investigation on taxonomical issues, for example on Empidoidae flies (Daugeron, in prep.) and Ichneumonidae wasps with possible new species for science. Molecular analyses (DNA barcoding) confirm an unexpected diversity in various Aculeata families (Schmid-Egger and Schmidt 2021) and parasitoid wasps of the genus *Brachygaster* Leach, 1815 (Evaniidae). One specimen of Pseudoscorpions (Arachnida) discovered in the estate is also potential new species for science.

### The Bois de Bouis' insect diversity compared to other arthropods inventories in France and Belgium

**Figure 7.**
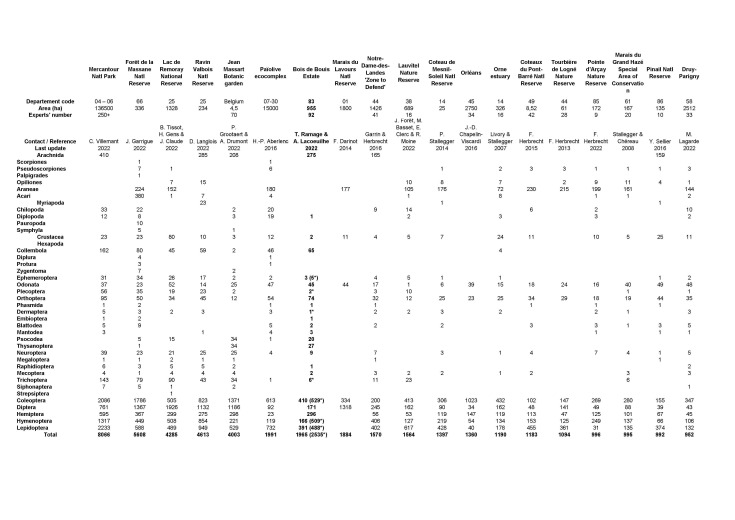
The main arthropods inventories in France and Belgium (number of species). Legend. * : diversity with morphospecies included. In bold: Bois de Bouis estate. Available as xls sheet in Suppl. material 1.

During the last 20 years, several comprehensive arthropod surveys, or ATBIs, have been realised in France and Belgium (Livory and Stallegger 2007, Stallegger and Chéreau 2008, Herbrecht 2013, Stallegger 2014, Darinot, F (coord.) 2014, Herbrecht et al. 2015, Ichter et al. 2018, Forêt et al. 2022, Grootaert and Drumont 2022, Ichter et al. 2022). We present here for the first time, a summary of the most relevant initiatives with species richness for each order (see Fig. 7 in png format or download as xls sheet in Suppl. material 1). Due to a great variety of areas, climates and ecosystems, the results of the different inventories cannot be directly compared. Apart from natural factors, the success, in particular species richness, of an arthropod survey also depends on the time invested and the diversity of sampling methods and specialists.

To illustrate the complex task of an exhaustive inventory, we can cite the Lac de Remoray National Reserve in the region Bourgogne-Franche-Comté, one of the best studied sites in France. As an example, Diptera diversity (1,926 species) represents 48% of the total number of insects inventoried in the Reserve while representing 20% of insect diversity in France. On the other hand, the Hymenoptera surveyed in the Reserve represents 13% (508 species) of Remoray's insect diversity against 20% at the national level. Experts consider that at least 1900 species of Hymenoptera can be expected on this site.

The Bois de Bouis estate, with 2458 species (including morphospecies), is the seventh most important inventory in terms of arthropod diversity in spite of much lower sampling intensity. Most of the specimens of Coleoptera, Diptera and Hymenoptera were collected by two Malaise traps set over several months in 2014 and 2015 (see sampling method). The other groups were mostly sampled through opportunistic surveys by individual experts.

The results on species richness are noteworthy considering a much lower sampling intensity and duration than other inventories (e.g. Mercantour National Park, Massane National Reserve, Bois de Païolive). Several insect orders like Coleoptera and, in particular, Hymenoptera are very diverse. Others like Diptera are not reflecting the actual diversity mainly due to the lack of specialists to study them.

### Can we call it an All taxa biodiversity inventory ?

The concept of an All Taxa Biodiversity Inventory was invented by the American ecologist, Daniel Janzen, for a project in Costa Rica and implemented for the first time in the Great Smoky Mountains National Park in the USA (Janzen and Hallwachs 1994, Mauz 2011). It can be defined as a comprehensive survey of the species living in a natural (or semi-natural) area, including data on their environment, their abundance, behaviour and genetic diversity (Deharveng et al. 2015, Ichter et al. 2022).

In 2017, the MNHN and the Mercantour National Park reviewed the major ATBIs worldwide, with particular emphasis on the initiatives in France. The final report co-authored by 12 experts proposed the following criteria to define an ATBI (Ichter et al. 2018):

- the diversity of taxonomic groups inventoried. An ATBI aims to study as many taxonomic groups as possible (including so-called "cryptic" biological compartments, such as arthropods, non-vascular flora and fungi);
- coordinated project management and sharing of knowledge. An ATBI implies coordination of the inventories (either centralised or distributed amongst the participants), data and specimens management and the dissemination of results;
- improving scientific knowledge. An ATBI aims to advance knowledge of taxonomy, biogeography and ecology. This implies significant sampling pressure and the collection of information on the spatial and temporal distribution of species, their habitats and, where applicable, their abundance and life history traits;
- a geographically coherent territory.

We argue that the work carried out on the Bois de Bouis estate responds to the above-mentioned criteria and can be considered an All taxa biodiversity inventory. The main arguments are: 1) significant sampling efforts over a large period (most groups have been sampled at least twice), 2) a wide variety of taxonomic groups (including cryptic groups such as Algae, Lichens, Nematodes, Aranea or Hymenoptera), 3) a high level of data quality and dissemination and 4) significant scientific results (Rault et al. 2015, Kehlmaier et al. 2019, Tillier 2019).

However, the biodiversity inventory is far from being comprehensive, especially for lesser-known groups like Flies, Worms or Moths. Most Fungi (except Lichens) were not surveyed during the project.

### Habitat type mapping

Natural habitats survey made it possible to identify 65 dominant habitats according to the EUNIS typology (European Topic Centre On Biological Diversity 2008). Several habitats are of community interest including rock slabs, Mediterranean siliceous grasslands, temporary Mediterranean ponds, Serapia grasslands, cork-oak woodlands, Mediterranean riparian forests, stone and mesogean pine forests (Charles and Viry 2015, Gourdain et al. 2017, Mistarz and Latour 2019, Latour et al. 2019).

In 2009, a first study on the habitats of the Bois de Bouis estate was carried out (Baret 2009). The whole site has been mapped at a scale of 1:5000 (Savio 2015). At this scale, the main issue was mapping habitat mosaics, when two or more vegetation types are found in close proximity, each occupying small areas. These repeating patterns can be explained by vegetation dynamics (communities substitute for each other over time) or by topographical factors due to micromorphology (Ichter et al. 2014a). Habitat mosaics represent more than half of the site (435 hectares).

**Figure 8.**
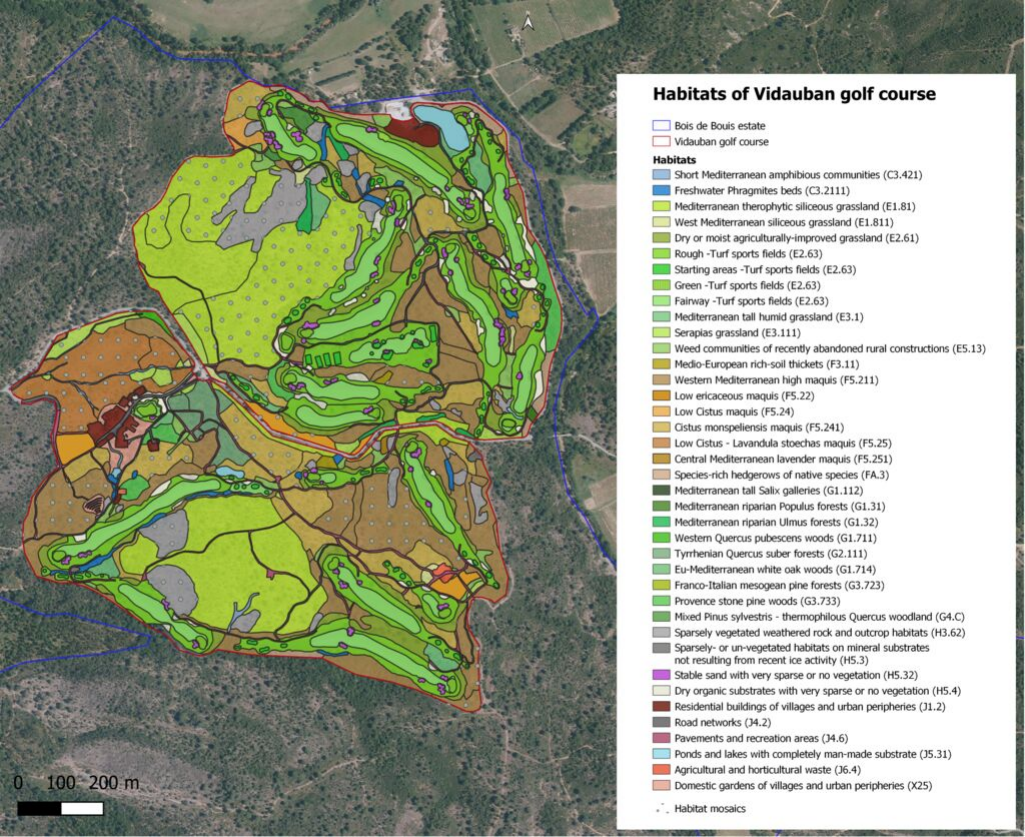
Last update (2021) of the Vidauban golf course habitat mapping at a scale of 1:5000 using EUNIS habitat typology.

Fig. 8 presents the golf course and estate habitat mapping and Fig. 9 presents the Bois de Bouis estate habitats mapping, both at a scale of 1:5000 using EUNIS habitat typology. In total, for the 830 hectares, 1244 habitat polygons were created.

### Conclusion

The Bois de Bouis estate inventory is one of a kind for several reasons. First the duration; it is not common to conduct and finance a 10-year survey on private land. Secondly, the scope of the inventories; biodiversity assessments are usually limited to a small number of well-known groups. Finally, the scientific outreach; the specimens collected and barcoded will allow taxonomic advances on poorly-known groups (e.g. Ichneumonidae) and more generally for taxonomic revisions whose scope goes beyond the initial objectives of the inventory and the perimeter of the Plaine des Maures.

## Supplementary Material

153F3E07-1F86-5EB4-95DF-3655FDFA6F80 10.3897/BDJ.11.e103280.suppl1 8240884 Supplementary material 1 Results of the main arthropods inventory in France and Belgium (ENG+FR) Data type Table Brief description This table presents a summary of the most relevant arthropod surveys, or ATBIs, in France and Belgium with species richness for each order. File: oo_818103.xls https://binary.pensoft.net/file/818103 T. Ramage (coord.)

## Figures and Tables

**Figure 9a.**
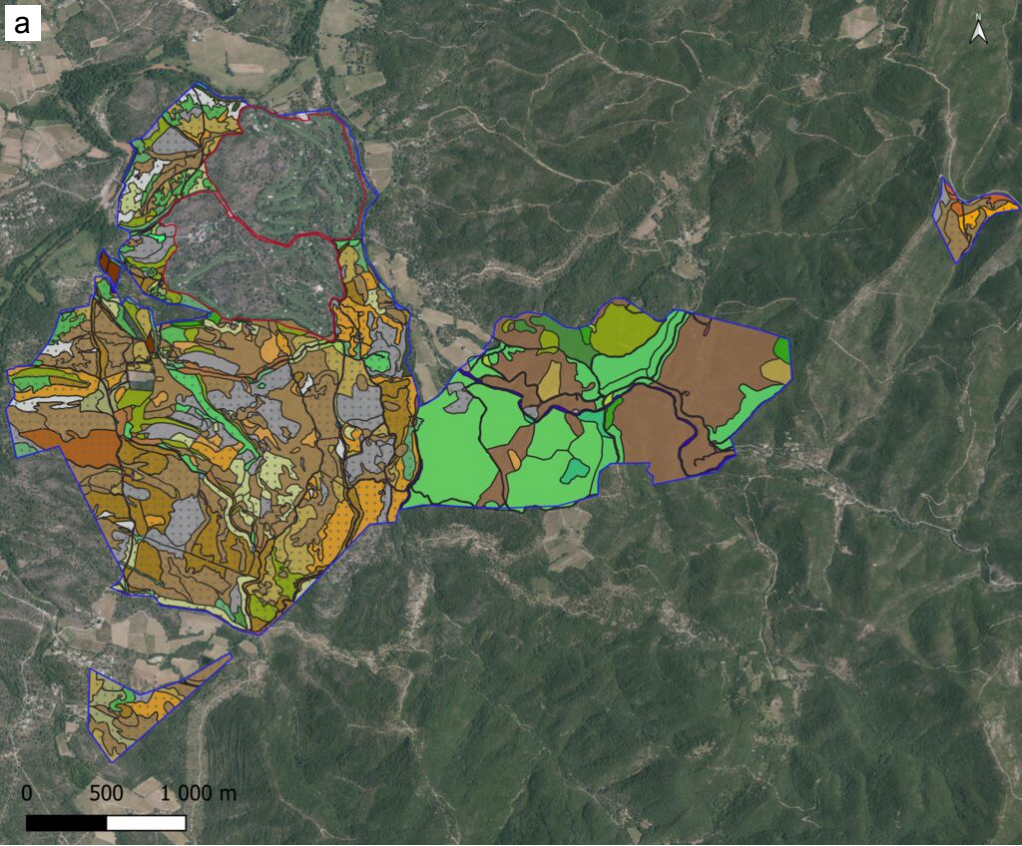
Last update (2017) of Bois de Bouis estate habitat mapping at a scale of 1:5000 using EUNIS habitat typology;

**Figure 9b.**
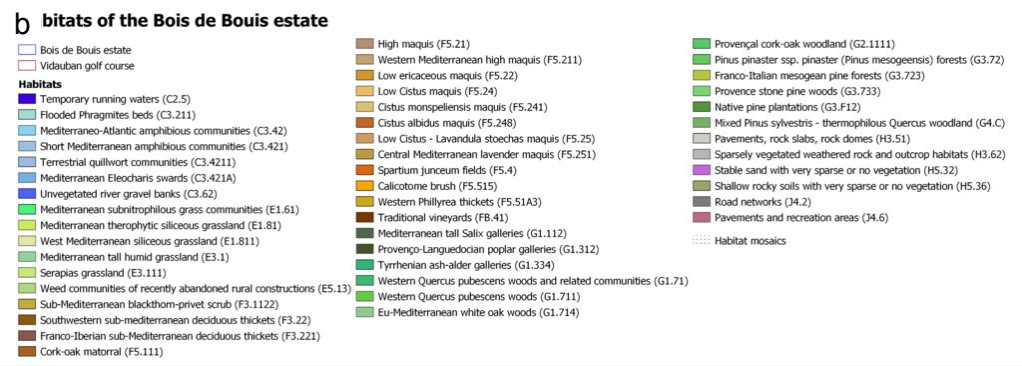
Legend of the Bois de Bouis Habitat mapping using EUNIS habitat typology.

**Table 1.** Proportion of metropolitan French species occurring in the Bois de Bouis estate.

			Species in France	Species in Bois de Bouis	Proportion
Fungi			24,497	N/A	N/A
	including Lichens		3,165	79	2.49%
Algae (sensu lato)			2,391	17	0.71%
Plants			10,113	1,049	10.46%
	Bryophytes		1,264	87	6.88%
	Angiosperms		7,625	933	12.24%
	Gymnosperms		73	6	8.22%
	Pteridophytes		179	23	12.85%
Animalia			48,746	2,003	4.10%
	Worms		1,376	1	0.01%
	Chordate		857	175	0.20%
		Birds	486	115	23.66%
		Fishes	81	3	3.70%
		Reptiles	41	17	41.46%
		Amphibians	43	9	20.93%
		Mammals	206	31	15.05%
	Mollucs		700	32	4.57%
	Rotifers		473	N/A	N/A
	Arthropods		45,191	1,796	3.97%
		Insects	39,447	1,537	3.90%
		Arachnids	3,481	228	6.55%
		Crustaceans	833	2	0.24%
		Myriapods	524	1	0.19%
		Entognatha	906	28	3.09%
	Tardigrades		67	N/A	N/A
Bacteria			169	N/A	N/A
Protozoa			525	N/A	N/A
Chromista			1,396	N/A	N/A
TOTAL			192,765	3,160	0.02%
